# Experimental non-alcoholic fatty liver disease causes regional liver functional deficits as measured by the capacity for galactose metabolism while whole liver function is preserved

**DOI:** 10.1186/s12876-022-02574-6

**Published:** 2022-12-27

**Authors:** Peter Lykke Eriksen, Karen Louise Thomsen, Stephen Hamilton-Dutoit, DMSc Hendrik Vilstrup, Michael Sørensen

**Affiliations:** 1 Department of Hepatology and Gastroenterology, Aarhus University Hospital, Palle Juul Jensens Boulevard 99, 8200 Aarhus N, Denmark; 2 Department of Internal Medicine, Randers Regional Hospital, Skovlyvej 15, 8930 Randers, Denmark; 3 Department of Pathology, Aarhus University Hospital, Palle Juul Jensens Boulevard 99, 8200 Aarhus N, Denmark; 4 Department of Nuclear Medicine & PET, Aarhus University Hospital, Palle Juul Jensens Boulevard 99, 8200 Aarhus N, Denmark; 5 Department of Internal Medicine, Viborg Regional Hospital, Heibergs Alle 5A, 8800 Viborg, Denmark

**Keywords:** Non alcoholic fatty liver disease, Experimental animal model, Positron emission tomography, Metabolic liver function

## Abstract

**Background:**

Increasing incidence of non-alcoholic fatty liver disease (NAFLD) calls for improved understanding of how the disease affects metabolic liver function.

**Aims:**

To investigate in vivo effects of different NAFLD stages on metabolic liver function, quantified as regional and total capacity for galactose metabolism in a NAFLD model.

**Methods:**

Male Sprague Dawley rats were fed a high-fat, high-cholesterol diet for 1 or 12 weeks, modelling early or late NAFLD, respectively. Each NAFLD group (n = 8 each) had a control group on standard chow (n = 8 each). Metabolic liver function was assessed by 2-[^18^F]fluoro‐2‐deoxy‐D-galactose positron emission tomography; regional galactose metabolism was assessed as standardised uptake value (SUV). Liver tissue was harvested for histology and fat quantification.

**Results:**

Early NAFLD had median 18% fat by liver volume. Late NAFLD had median 32% fat and varying features of non-alcoholic steatohepatitis (NASH). Median SUV reflecting regional galactose metabolism was reduced in early NAFLD (9.8) and more so in late NAFLD (7.4; p = 0.02), both significantly lower than in controls (12.5). In early NAFLD, lower SUV was quantitatively explained by fat infiltration. In late NAFLD, the SUV decrease was beyond that attributable to fat; probably related to structural NASH features. Total capacity for galactose elimination was intact in both groups, which in late NAFLD was attained by increased fat-free liver mass to 21 g, versus 15 g in early NAFLD and controls (both p ≤ 0.002).

**Conclusion:**

Regional metabolic liver function was compromised in NAFLD by fat infiltration and structural changes. Still, whole liver metabolic function was preserved in late NAFLD by a marked increase in the fat-free liver mass.

**Supplementary Information:**

The online version contains supplementary material available at 10.1186/s12876-022-02574-6.

## Background

The increasing prevalence of non-alcoholic fatty liver disease (NAFLD) underlines the need for improved understanding of how the disease affects the functional integrity of the liver [1, 2]. Hepatic tissue lesions in NAFLD are caused by fat accumulation (non-alcoholic fatty liver, NAFL), and in non-alcoholic steatohepatitis (NASH) by further damage associated with inflammation, hepatocyte ballooning and liver cell death. As the disease progresses, hepatic scarring by fibrosis develops [3]. Previously, the clinical and pathophysiological perception was that liver function remains largely unaffected until the most advanced stages of NAFLD with cirrhosis. However, accumulating evidence indicates that liver function in NAFLD is compromised, even in the early stages of the disease [4–8].

The structural abnormalities of the liver parenchyma found in NAFLD may explain the compromised liver tissue function. Thus, hepatic fat may both displace functional liver tissue and disturb hepatocyte function by lipotoxic effects [9]. Fibrosis leads to additional disturbance of the liver parenchyma and hepatic function could be further compromised by hepatocyte death. Positron emission tomography (PET) imaging studies using the tracer [^18^F]fluoro-2-deoxy-D-galactose (^18^F-FDGal), which is specifically metabolised by galactokinase in hepatocytes, can be used to study the capacity for hepatic galactose metabolism which is a validated measure of overall metabolic liver function [10–12]. From these studies, it is evident that in established cirrhosis, in which structural abnormalities including fibrosis are widespread, regional metabolic function is markedly reduced and heterogeneously distributed throughout the liver tissue [12, 13]. Interestingly, similar changes were observed in patients with NASH, but not in patients with NAFL, in whom liver function was unaffected when correcting for the mass effect of fat infiltration [8].

All in all, NAFLD may be expected to be deleterious to regional and whole liver metabolic function, further harm being inflicted by the structural changes of more advanced NAFLD. The aim of the present study was to investigate the effect of different stages of experimental NAFLD on regional and whole liver metabolic function. For this purpose, rats were fed a Western-style high-fat high-cholesterol (HFHC) diet for either 1 or 12 weeks, modelling early and late NAFLD, respectively. Metabolic liver function was investigated by ^18^F-FDGal micro PET and compared with ^18^F-FDGal autoradiography, histology, and hepatic fat quantification.

## Methods

### Animals

Male Sprague Dawley rats (Taconic Biosciences, Ejby, Denmark) were housed at 21 ± 2 °C with a 12-hour artificial light cycle. The animals were kept in pairs with free access to water and acclimatised on normal chow, before each cage was randomised to either HFHC (D09052204, Research Diets, New Brunswick, NJ) or standard diet (Altromin #1324, Brogaarden, Denmark) ad libitum. The HFHC diet contained 2 g cholesterol per 100 g diet, with an energy contribution of 15% carbohydrates, 20% protein, and 65% fat [14]. In the standard diet, the energy contribution was 65% carbohydrates, 24% protein, and 11% fat (ingredients: cocoa butter, casein, maltodextrin, soybean oil, sucrose, L-cystein in addition to cholesterol, sodium cholate, choline bitartrate, minerals, vitamins and cellulose). We aimed at 1-week and 12-weeks animals having similar body weights at the time of investigation. For this reason, and based on pilot experiments, 12-week animals had a lower baseline weight (median weight 223 g, range 196–283; approximately age 7 weeks at inclusion) than 1-week animals (median weight 398 g, range, 366–419; approximate age 13 weeks at inclusion). The number of animals in each group was based on experiences from previous experiments. No animals were excluded.

### Experimental design

**Fig. 1:**
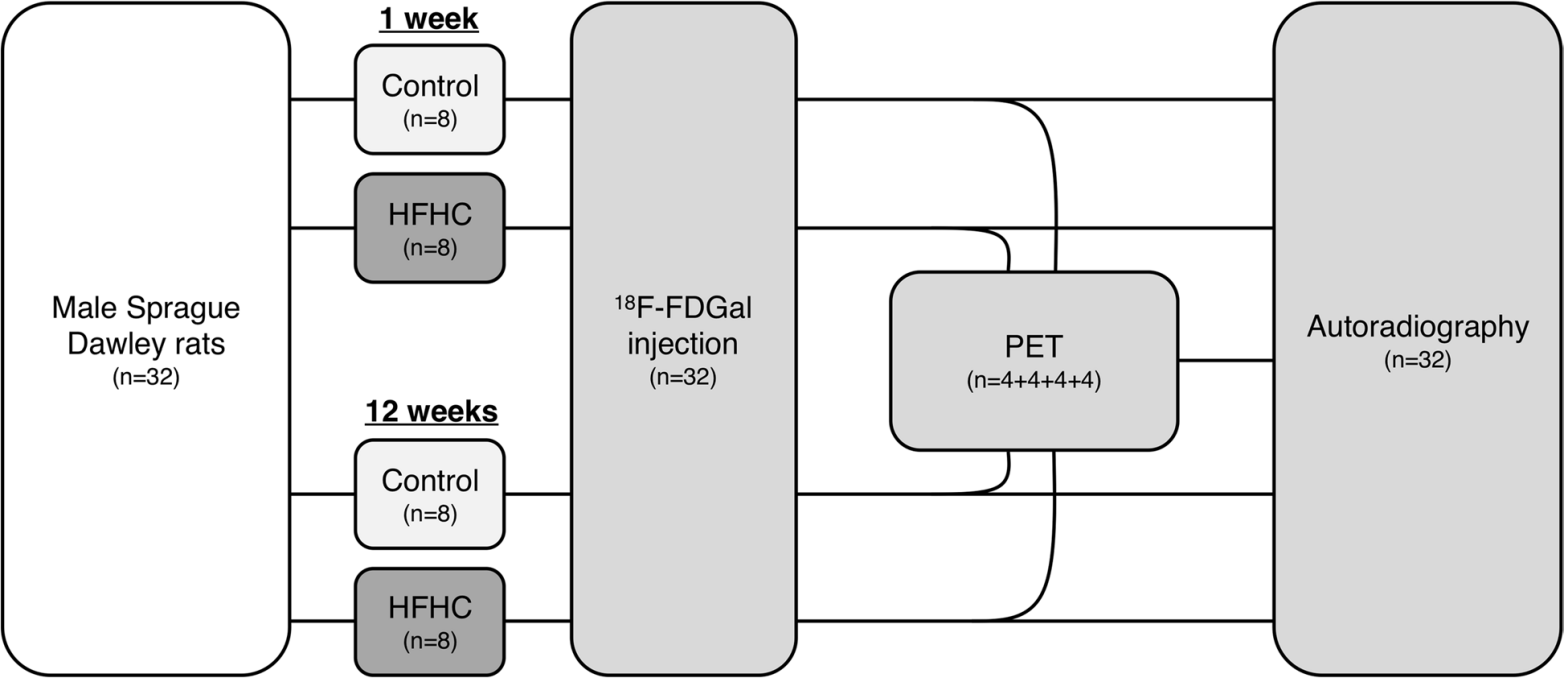
Study design Rats were fed a high-fat high-cholesterol (HFHC) diet for one week (early non-alcoholic fatty liver disease, NAFLD) or twelve weeks (late NAFLD) with corresponding control groups on a standard diet. Following an intravenous bolus injection of 2-[^18^F]fluoro-2-deoxy-D-galactose (^18^F-FDGal), half of the animals in each of the four groups were investigated in vivo by a dynamic positron emission tomography (PET) recording and all animal livers by *post mortem* autoradiography

Figure 1 shows the experimental design of the study. In short, the study comprised two parallel experiments. Each experiment had an HFHC group (n = 8) and a control group on standard chow (n = 8). Each animal was studied once; either one week after start of diet in *Substudy 1* or 12 weeks after start of diet in *Substudy 2*. One-week HFHC animals were intended to model early NAFLD with bland steatosis, whilst 12-weeks HFHC animals were intended to model late NAFLD with features of NASH/fibrosis. All animals were studied with ^18^F-FDGal, which was injected in vivo. In each of the four groups, half of the animals (n = 4) were studied with ^18^F-FDGal micro PET, ^18^F-FDGal autoradiography, and histology, and the other half (n = 4) were studied with ^18^F-FDGal autoradiography and histology.

Animals within each group were investigated in pairs by cage. On the day of investigations, anaesthesia was induced with isoflurane 2–3% (Forene®, Abbott Laboratories, Gentofte, Denmark) and both animals were kept anaesthetised with inhalation of isoflurane delivered through a head mask throughout the procedures. After placement of an intravenous (IV) line in a tail vein, both animals were injected IV with a bolus of 15 MBq (median 14.4 (range, 6.6–16.9) ^18^F-FDGal, approximate volume of 0.8 mL including a 0.2 mL sodium chloride flush), produced at the in-house radiochemistry facilities at the Department of Nuclear Medicine & PET, Aarhus University Hospital [15]. Because of size limitations of the micro PET scanner, the smaller of the two animals in each cage was placed in the scanner (Mediso nanoScan PET/MRI, Budapest, Hungary) and the other was studied with ^18^F-FDGal autoradiography alone.

After 50 min, comprising a 30 min dynamic PET-scan and an approximately 20 min MRI transmission scan for attenuation correction of the PET data, blood was drawn from both animals via puncture of the heart under continuous anaesthesia with inhalation of isoflurane. While still anaesthetised, the livers were rapidly removed and weighed and animals were euthanatised by cervical dislocation. The left median hepatic lobe was immediately frozen in isopentane at − 40° for approximately 30 s and thereafter cryostat cut into 10 μm sections for autoradiography and histology. Also, slices of liver tissue were harvested and snap frozen in liquid nitrogen for measurement of hepatic fat content.

### ^*18*^*F-FDGal PET analysis*

PET scans were corrected for radioactive decay to the time of injection time and reconstructed using Tera-Tomo 3D full detector model, with 4 iterations, 6 subsets, a voxel size of 0.4 mm, and a matrix of 160 × 155 × 236.

We used PMOD software version 3.7 (PMOD Technologies Ltd., Zürich, Switzerland) for analysis of the PET images. The liver volume was determined using the Isocontour tool with a dynamic threshold of 50% of regional liver tissue ^18^F-FDGal uptake in a smaller volume-of-interest (VOI) placed in the, easily identifiable, right median liver lobe (see below). The total amount of ^18^F‐FDGal accumulated in the total liver volume was used to express the whole liver metabolic function in terms of percentage of injected dose (%ID).

The regional VOI in the right median liver lobe (approximately 0.1 mL liver tissue) was used to calculate the standardised uptake value (SUV) of ^18^F-FDGal as mean liver tissue radioactivity concentration in the VOI (kBq/mL liver tissue), recorded from 10 to 30 min after ^18^F‐FDGal injection, divided by the amount of radioactivity injected per kilogram body weight (kBq/kg). A fat mass corrected SUV was calculated from the regional liver tissue ^18^F‐FDGal uptake in the VOI subtracted the fat fraction (volume%, see below). Functional tissue heterogeneity was defined as the SUV coefficient of variation (COV_SUV_), calculated as the standard deviation of the SUV divided by the mean SUV.

### Autoradiography

Autoradiography was used for direct comparison between liver tissue ^18^F-FDGal accumulation and histology. The cryostat cut liver slices were air dried and exposed on a Fuji imaging plate (Bas-IP SR 2025; Fuji Photo Film Co., Ltd., Tokyo, Japan) for 2 h to image the radioactivity of the hepatic ^18^F-FDGal uptake, along with in-house manufactured, radioactivity concentration standards. The imaging plate was analysed on a BAS 5000 scanner (Fuji Photo Film Co., Ltd., Tokyo, Japan) yielding images with a resolution of 25 μm. Analysis of the autoradiography data was performed using Fiji for ImageJ 2.0 [16, 17] and the plug-in ISAAC Manager [18]. After generating a calibration curve via the ‘Rodbard fit’ from the known standards, corrected for radioactive decay to the time of injection time, tissue radioactivity (kBq/mL) was measured within a 7.84 mm^2^ square region of interest, free of larger vessels and portal tracts. Results were based on the mean of three measurements, on three different slides from each liver, corrected for the injected dose and expressed as %ID/mL liver tissue. A coefficient of variation was calculated to describe the functional tissue heterogeneity.

### Histology

Liver sections were stained with haematoxylin and eosin, and Masson’s trichrome, and evaluated by an expert liver histopathologist (SHD) blinded to the study. All slices were scored for steatosis, activity, fibrosis (SAF) [3], NAFLD Activity Score (NAS), and Kleiner Fibrosis Score (F0–4) [19].

### Liver fat quantification

Hepatic fat was extracted from approximately 1 g of snap frozen liver tissue, based on extraction of fat by the Folch method [20, 21]. Liver tissue fat was weighed (g) and the fat infiltration was expressed as liver fat volume fraction (%vol) based on a density of 0.9 g/mL for fat and 1.05 g/mL for non-fat liver. These results were used to calculate a fat-corrected liver weight, a fat-corrected SUV (from PET), and a fat-corrected radioactivity concentration (%ID/mL fat-free liver tissue; autoradiography).

### Biochemical analysis

All biochemical blood analyses except insulin were determined using routine analyses with accredited laboratory assays at the Department of Clinical Biochemistry, Aarhus University Hospital. Alanine aminotransferase, bilirubin, albumin, cholesterol, triglycerides, and glucose were analysed with Cobas E601 (Roche, Basel, Switzerland) and prothrombine-proconvertin ratio with Sysmex Cs2100i (Siemens Healthineers, Erlangen, Germany). Insulin was measured in house by use of the Ultra Sensitive ELISA kit (Biorbyt Ltd., Cambridge, UK).

### Statistical analyses

Early and late NAFLD substudies were conducted as parallel experiments with their own individual control groups. Statistical comparisons between the two experimental NAFLD groups were performed via variables normalised to the median value of their respective control group. Data were analysed using Stata v.14.2 (StataCorp, College Station, TX) by the Wilcoxon–Mann–Whitney non-parametric test for continuous variables and by the Fisher exact test for ordinal variables. Data are presented as median (range). A two-sided p-value < 0.05 was considered statistically significant.

## Results

### Animal characteristics

As shown in Table 1 and Supplementary Figure 1, body weight on the day of investigations was lower in the early NAFLD animals and their controls, with no significant difference between these (p = 0.4), when compared with late NAFLD animals and their controls. Body weight of the late NAFLD animals was ~ 10% greater compared with their control group (p = 0.07).

**Table 1:** Biochemistry, liver morphometrics, histology and ^18^F-FDGal PET and autoradiography data

	1 week		12 weeks	
	Controls	Early NAFLD	Controls	Late NAFLD
*Basic characteristics*	*n = 8*	*n = 8*	*n = 8*	*n = 8*
Body wt (g)	423 (399–436)	428 (386–444)	482 (426–551)	533 (452–620)
ALT (U/L)	35 (30–49)	62 (39–80)**	37 (34–55)	76 (51–110)**
Albumin (g/L)	15 (12–16)	15 (14–16)	14 (13–15)	13 (11–15)*
Bilirubin (µmol/L)	< 2	< 2	< 2	< 2
Coagulation factors II, VII, X (PP)	0.31 (0.27–0.34)	0.31 (0.29–0.33)	0.31 (0.29–0.32)	0.33 (0.30–0.39)
Cholesterol (mmol/L)	1.6 (1.5–2.2)	3.1 (2.6–3.6)***	1.7 (1.5–2.4)	3.5 (2.7–5.1)***
Triglycerides (mmol/L)	1.4 (0.6–2.8)	1.9 (0.7–2.6)	1.2 (0.7–1.8)	0.9 (0.5–1.6)
Blood glucose (mmol/L)	8.2 (6.5–11.3)	7.7 (6.0–9.8)	8.8 (7.4–14.5)	8.2 (7.1–9.3)
Insulin (ng/mL)	0.6 (0.2–1.2)	0.8 (0.4–2.7)	1.6 (0.9–2.8)	0.9 (0.2–2.5)
*Liver morphometrics*	*n = 8*	*n = 8*	*n = 8*	*n = 8*
Liver wt (g)	15.1 (14.2–16.3)	18.7 (14.8–19.6)**	15.6 (13.7–16.5)	29.6 (20.6–34.6)***
Liver volume (mL)	14.7 (13.9–15.4)	19.1 (14.2–20.2)	14.5 (13.4–17.2)	27.1 (21.5–31.7)*
Liver fat fraction (% volume)	5.2 (4.9–5.3)	18.3 (15.5–21.7)***	5.1 (4.7–5.5)	31.7 (27.4–41.4)***
Fat-corrected liver wt (g)	14.4 (13.5–15.5)	15.4 (12.8–16.0)*	14.9 (13.1–15.8)	21.1 (15.6–22.3)**
Fat-corrected liver wt/body wt (%)	3.5 (3.1–3.8)	3.6 (3.3–3.8)	3.1 (2.8–3.2)	3.6 (3.4–4.2)***
*Liver histology*	*n = 8*	*n = 8*	*n = 8*	*n = 8*
Steatosis^a^, n_0 / 3_	8 / 0	0 / 8***	8 / 0	0 / 8***
Inflammation^a^, n_1 / 2_	1 / 0	3 / 0	0 / 0	6 / 0**
Ballooning^a^, n_1 / 2_	0 / 0	0 / 0	0 / 0	2 / 1
Fibrosis score^b^, n_1a / 1b_	0 / 0	0 / 0	0 / 0	2 / 1
NAS score	0 (0–1)	3 (3–4)***	0	4 (3–6)***
^*18*^*F-FDGal PET-measurements*	*n = 4*	*n = 4*	*n = 4*	*n = 4*
SUV, right median lobe	11.9 (11.3–12.6)	9.8 (9.2–10.4)*	13.1 (10.8–13.7)	7.4 (6.3–9.3)*
Fat-corrected SUV	12.6 (12.0–13.3)	12.0 (11.4–12.7)	13.8 (11.3–14.4)	11.2 (8.9–12.8)
COV_SUV_ (%)	6.4 (5.3–7.8)	7.2 (6.2–8.2)	6.8 (5.0–7.2)	8.7 (7.3–9.8)*
Total accumulated (%ID)	34 (33–35)	35 (30–37)	34 (27–37)	35 (28–40)
^*18*^*F-FDGal Autoradiography*	*n = 8*	*n = 8*	*n = 8*	*n = 8*
Radioactivity concentration (%ID/mL)	1.12 (0.95–1.38)	0.91 (0.72–1.25)	1.07 (0.81–1.29)	0.62 (0.52–1.00)**
Fat-corrected radioactivity conc. (%ID/mL)	1.19 (1.00–1.45)	1.14 (0.85–1.48)	1.13 (0.86–1.36)	0.94 (0.76–1.38)
COV (%)	24 (22–27)	26 (24–33)	30 (17–33)	33 (24–46)*

Values are given as median (range). NAFLD, non-alcoholic fatty liver disease; wt, weight; ALT, alanine aminotransferase; PP, Prothrombine-proconvertin ratio; NAS: Non-alcoholic fatty liver disease activity score; %ID, percentage of the injected dose of ^18^F-FDGal accumulated in the liver; SUV: standardised uptake value; COV: coefficient of variation; ^a^: based on steatosis activity fibrosis (SAF) score, ^b^: based on Kleiner fibrosis score

*P* when compared with controls: * < 0.05, ** < 0.01, *** < 0.001

Plasma concentrations of alanine aminotransferase and cholesterol were higher in NAFLD animals compared with their respective control groups (p < 0.01). The biochemical markers of liver function, bilirubin and coagulation factors, were comparable. For albumin, there was a small difference between late NAFLD (13 g/L) and their controls (14 g/L) (p = 0.03). There were no differences in plasma glucose, insulin, and triglycerides between the groups (Table 1).

In early NAFLD, the liver was more than 25% larger than in control animals (median 19 g vs. 15 g; p = 0.004); in late NAFLD, the liver weight was nearly doubled (median weight 30 g vs. 16 g; p < 0.001). The liver fat fraction in early NAFLD was almost fourfold higher than in controls (18% vs. 5%; p < 0.001); in late NAFLD, it was more than sixfold higher (32% vs. 5%; p < 0.001). The fat-corrected liver weight was slightly increased by 7% in early NAFLD (p = 0.04), whereas it was increased by 40% in late NAFLD compared with controls (p = 0.001). This was also reflected by a significantly increase in the fat-corrected liver weight to body weight ratio in late NAFLD animals only (Table 1). Both the uncorrected and the fat-corrected liver weight and the fat-corrected liver weight to body weight ratio was greater in late, compared with early NAFLD animals (p ≤ 0.002).

### ^*18*^*F-FDGal PET*

**Fig. 2:**
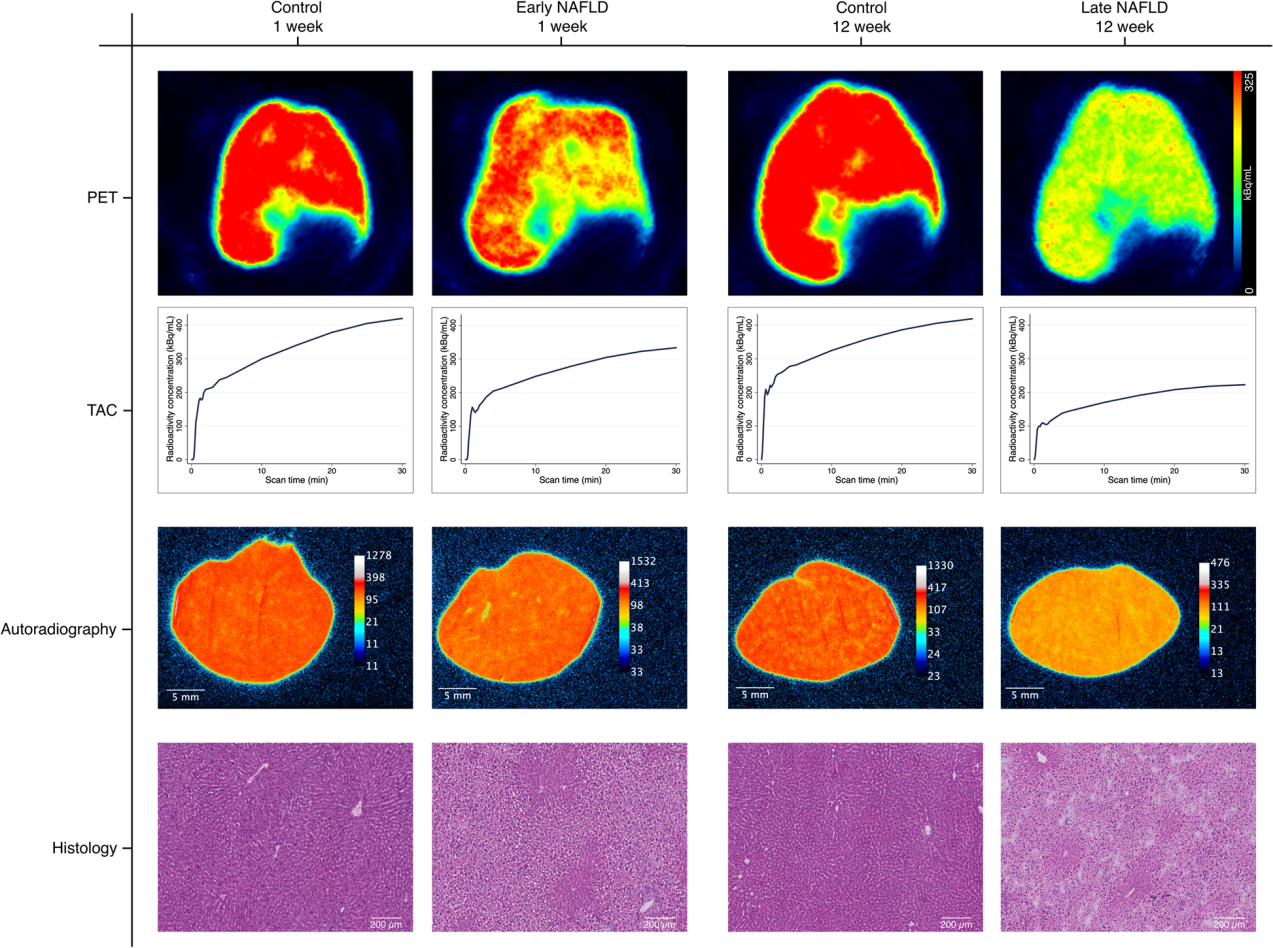
^18^F-FDGal PET scan, autoradiography and histology Representative examples from each study group (controls and high-fat high-cholesterol, HFHC) of the trans axial summed images from 10–30 min of the dynamic positron emission tomography recordings and time activity courses of the radioactivity concentrations in the liver tissue (TAC, smoothing parameter 0.20 [30]) following an intravenous bolus injection of 2-[^18^F]fluoro-2-deoxy-D-galactose and corresponding autoradiography and haematoxylin and eosin stained histology sections

Regional metabolic liver function reflected by the accumulation of ^18^F-FDGal in liver tissue (kBq/mL liver tissue and SUV) during the PET recording was clearly and significantly decreased in NAFLD when compared with control animals; this was particularly apparent in late NAFLD (SUV 7.4 (6.3–9.3)), compared with early NAFLD animals (9.8 (9.2–10.4); p = 0.02) (Table 1; Fig. 2). When corrected for fat-infiltration, SUV was similar comparing early NAFLD and control animals (12.0 (11.4–12.7) vs. 12.6 (12.0–13.3); p = 0.25), but tended to be lower in late NAFLD animals, compared both with their controls (11.2 (8.9–12.8) vs. 13.8 (11.3–14.4); p = 0.08), and with early NAFLD animals (p = 0.08).

With regard to whole liver metabolic function, measured as the percentage of injected dose (%ID) accumulated in the whole liver, no difference was found between the four groups of NAFLD and control animals (~ 35%ID, p = 0.4; Table 1). Thus, the overall capacity of the liver for metabolising ^18^F-FDGal was intact and not compromised at either stage of NAFLD.

Visually, the late NAFLD livers exhibited a more heterogeneous distribution of ^18^F-FDGal accumulation than the early NAFLD livers. This was confirmed statistically by the higher COV_SUV_ for late NAFLD versus controls (median 8.7 vs. 6.8%, p = 0.02), whereas no significant difference was found between early NAFLD and controls (7.2 vs. 6.4%; p = 0.25). There was no statistically significant difference in COV_SUV_ between early and late NAFLD animals.

**Fig. 3:**
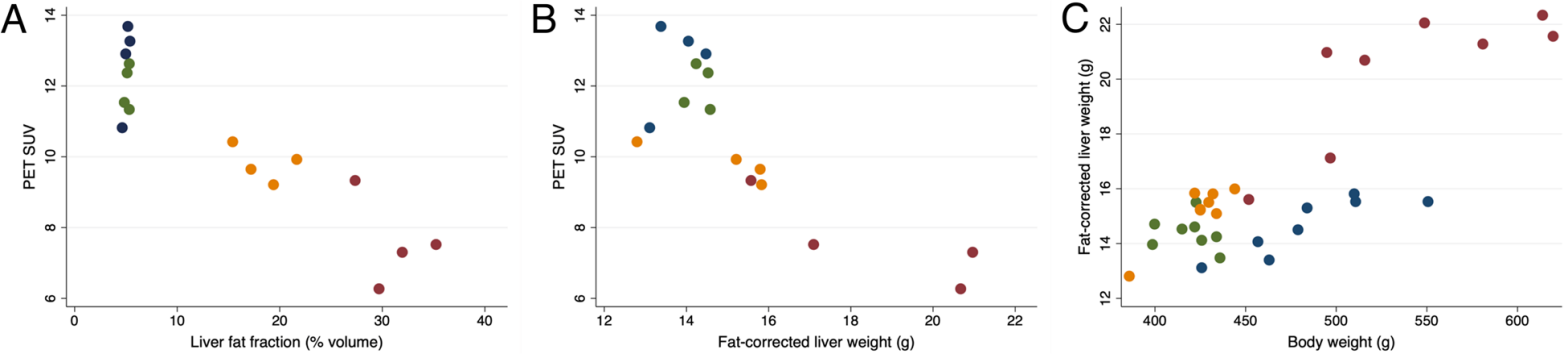
Relationship between ^18^F-FDGal PET SUV and liver fat, fat-corrected liver weight and bodyweight Relationship between liver accumulated 2-[^18^F]fluoro-2-deoxy-D-galactose measured by positron emission tomography (PET) and expressed as SUV, and liver fat volume fraction (A), fat-corrected liver weight (B) and body weight (C) in early (orange) and late (red) non-alcoholic fatty liver disease animals and their respective control groups (green and blue)

For the NAFLD animals, there was an inverse relationship between the local accumulation of ^18^F-FDGal in the liver, and the liver fat fraction (Fig. 3 A), indicating that fat infiltration diluted the ^18^F-FDGal PET signal. Similarly, greater fat-corrected liver weight was related to lower ^18^F-FDGal accumulation and to greater body weight (Fig. 3B + C). In late NAFLD animals, the fat-corrected liver mass increased more than body weight (Table 1; Fig. 3 C).

### Histology and autoradiography

All NAFLD animals had grade 3 steatosis. Three early, and six late NAFLD animals had liver inflammation. Ballooning and fibrosis grade 1 were observed in three of these six late NAFLD animals (Table 1). The control animals had no steatosis, inflammation, ballooning or fibrosis.

**Fig. 4:**
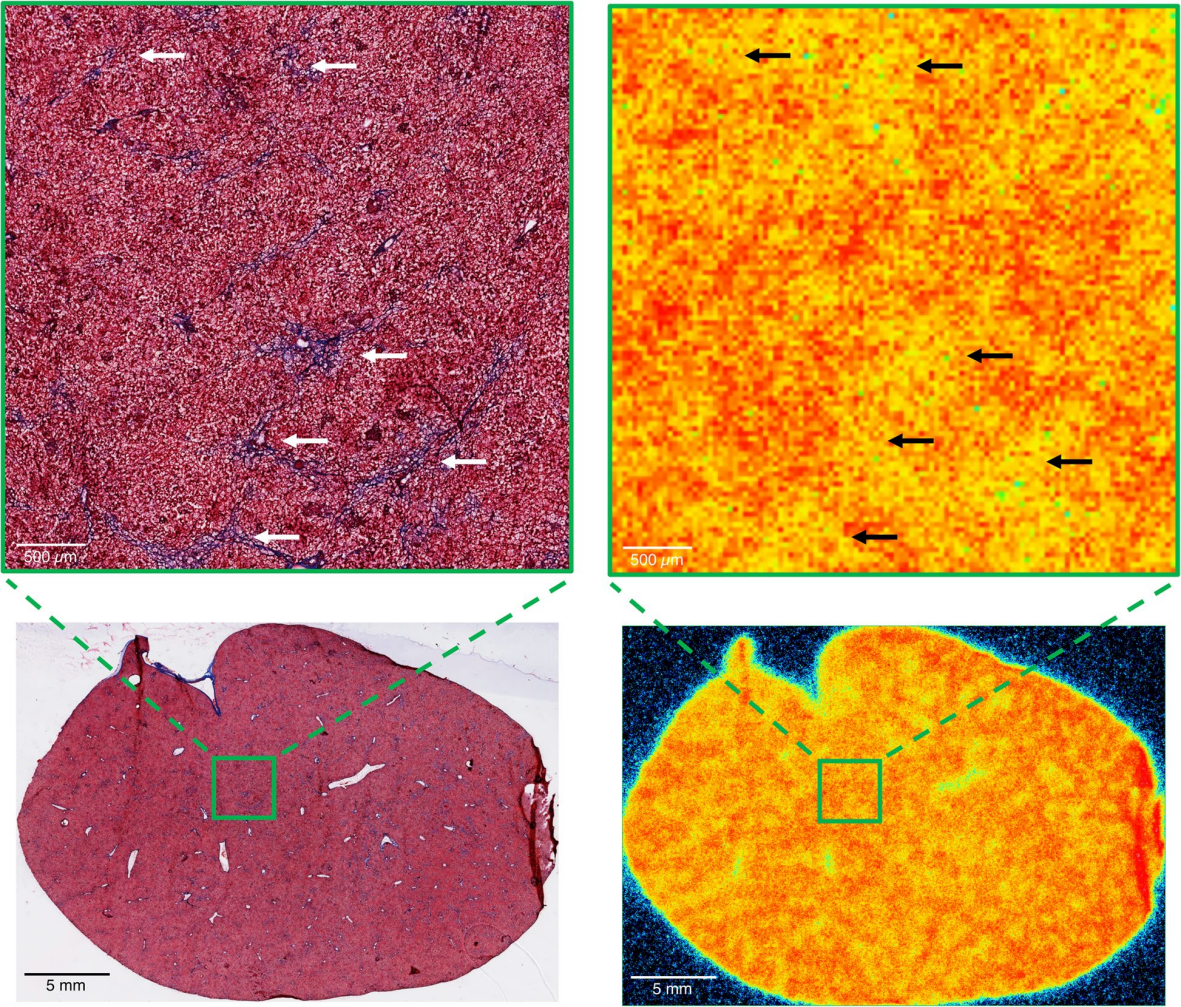
Fibrosis and ^18^F-FDGal liver tissue uptake Section of liver from a late non-alcoholic fatty liver disease animal with grade F1 fibrosis, Masson’s trichrome (MT) stained (left) and recorded for ^18^F-FDGal liver tissue uptake by autoradiography (right). Magnification of corresponding area (green box) is shown. Visually liver tissue fibrosis (stained blue on MT, indicated by white arrows) corresponded to lower 2-[^18^F]fluoro-2-deoxy-D-galactose liver tissue uptake (low uptake: yellow, indicated by black arrows vs. high uptake: red on autoradiography)

Autoradiography measurements of ^18^F-FDGal liver uptake (%ID/mL) correlated with the PET SUV (Supplementary Figure 2) and showed similar patterns, namely less uptake of ^18^F-FDGal in the NAFLD groups compared with controls. This was more pronounced (and only significant) for late NAFLD animals (p = 0.002); these also had a significantly higher COV than their controls (p = 0.02; Table 1). Again, as with the PET data, when correcting uptake for fat-infiltration, there was no difference between early NAFLD and controls (p = 0.92), whereas late NAFLD animals had lower fat-corrected tracer uptake compared with their controls (p = 0.09). Comparing early and late NAFLD animals, normalised to their respective control group, late NAFLD animals had significantly lower ^18^F-FDGal tracer uptake (p = 0.006), although the difference in fat-corrected tracer uptake and COV did not reach statistical significance (p ≥ 0.12). Visually, areas with fibrosis accumulated less ^18^F-FDGal than the surrounding tissue (Fig. 4). Fibrosis was mild and inconsistent; thus its functional consequence could not be quantified.

## Discussion

Our study shows that in a rodent model of diet-induced NAFLD, regional metabolic capacity of the liver was reduced, as measured by decreased regional accumulation of the galactose tracer ^18^F-FDGal in the liver tissue. Still, the total metabolic capacity for liver ^18^F-FDGal metabolism remained intact. In early NAFLD, fat infiltration in the liver accounted for the reduction in regional ^18^F-FDGal metabolism, whilst in late NAFLD there seemed to be a further reduction in regional ^18^F-FDGal metabolism, probably related to the structural changes associated with the disease. In late NAFLD, the maintenance of total hepatic galactose elimination capacity occurred by growth of functional liver tissue, manifested by increased fat-free liver weight. The PET measurements were validated by autoradiographic findings.

The functional and morphological changes in the early NAFLD livers were nearly completely explainable by the fat burden alone, since subtraction of the liver fat fraction resulted in near-normal regional liver function and liver mass. was different in livers from late NAFLD animals, in which fat-correction was not able to fully account for the reduced and more heterogeneous distribution of the metabolic liver function. This likely reflects the presence of injured hepatocytes (apoptotic or ballooned liver cells) and/or replacement of liver tissue by other non-fat tissue components such as inflammatory infiltrates and fibrosis, causing a further disturbance in the functional hepatic micromilieu. These results are consistent with the findings of our human study, in which NASH patients had reduced fat-corrected regional metabolic liver function, ascribable to the presence of ballooning and fibrosis [8]. Thus, both in experimental and human NAFLD, reduced regional accumulation of ^18^F-FDGal probably represents locally diluted or locally lost functional hepatocyte mass.

The rate-limiting step in hepatic metabolism of galactose and ^18^F-FDGal is phosphorylation by galactokinase and not perfusion [22]. The disturbed microcirculation with reduced sinusoidal perfusion that has been reported in NASH [23–25] is thus unlikely to affect the measurements presented here. If so, the local sinusoidal perfusion would have to be so low, that the area of the liver for all practical purposes would be considered non-functioning. The same would apply to other factors such as collagen deposition in the space of Disse [24], which might delay some hepatocytes in being exposed to the tracer, but this is also unlikely to affect the steady state metabolic trapping of tracer. Possible intracellular effects of NAFLD on galactokinase expression or enzyme properties, for example exerted by lipotoxicity and the inflammatory milieu [9, 26], have not been studied, but could be of importance, though galactokinase is regarded a constitutional and non-inducible enzyme [27].

An important observation, in both the present experimental study and our human study using ^18^F-FDGal PET and the galactose elimination capacity test [8], is that the total capacity for galactose metabolism remains intact even in late NAFLD/NASH, in spite of the reduction in galactose metabolism per mL liver tissue. This raises the question of how whole liver metabolic function is preserved. In early NAFLD, our present study shows that the fat mass of simple liver steatosis is simply added to an otherwise normal sized and functional liver. In contrast, in both late NAFLD animals and in NASH patients, there is a marked increase in fat-corrected liver mass. Thus, in later NAFLD stages, whole liver metabolic function seems to be maintained by growth of functional liver. This difference between early and late experimental NAFLD - and between human NAFL and NASH - may indicate that the two stages of NAFLD are actually different diseases with basically different influences on liver mass requirements. While our present study sheds light on the histological structural explanation of the findings in humans, it does not provide insights into this possible compensatory growth of the functional liver mass in late NAFLD. Liver size adaptation to metabolic demands is complex and its regulation remains largely unknown, but involves numerous cytokines, gut-derived growth factors, and hepatic blood flow in a complex interplay [28].

There are limitations to our study. First, sample size was small with only four animals in each of the PET groups, and the invasive nature of the study prevented a longitudinal study of individual animals. Despite the small sample size, differences between the groups with regard to the main outcomes studied were consistent and statistically significant. Moreover, the borderline significant results on reduced fat-corrected ^18^F-FDGal accumulation in late NAFLD may well be the result of the low number of observations; the group difference visually and numerically seemed convincing. Furthermore, caution should be used when extrapolating results from animal studies to humans and the diagnostic histological criteria for NASH in humans, which include the presence of liver cell ballooning, was met in only three out of eight late NAFLD animals. This is a well-known challenge using animal models of NAFLD [29]. Also, fibrosis was not a common finding among the animals, even after 12 weeks.

The observations on liver growth in late NAFLD rely on the correct measurement of hepatic fat. We used a well-established fat extraction method for this purpose and our conclusions are corroborated by a similar observation in our human study, in which fat was determined by magnetic resonance imaging proton density [8].

## Conclusion

In conclusion, our findings provide evidence that NAFLD causes local liver functional deficits while whole liver metabolic function is maintained. The regional deficit is accounted for by mass dilution of liver tissue with fat. In more advanced NAFLD, the local deficit was not fully accountable for by fat alone, and further structural changes and/or effects of the disease likely contributed. In advanced disease stages, our data suggest that the whole liver metabolic function may be preserved by the marked growth of fat-free liver mass. The mechanisms behind this functionally compensatory liver growth are not known.

## Supplementary Information

**Additional file 1: **Supplementary Figures 1 and 2.

## Data Availability

The PET data are available from the corresponding author (PLE) upon reasonable request.

## Abbreviations

COV: coefficient of variation
18F-FDGal: [18 F]fluoro-2-deoxy-D-galactose
HFHC: high-fat high-cholesterol
IV: intravenous
NAFL: non-alcoholic fatty liver
NAFLD: non-alcoholic fatty liver disease
NASH: non-alcoholic steatohepatitis
PET: positron emission tomography
SUV: standardised uptake values
VOI: volume-of-interest
%ID: percentage of injected dose
