# Correction: Experimental non-alcoholic fatty liver disease causes regional liver functional deficits as measured by the capacity for galactose metabolism while whole liver function is preserved

**DOI:** 10.1186/s12876-023-02654-1

**Published:** 2023-02-23

**Authors:** Peter Lykke Eriksen, Karen Louise Thomsen, Stephen Hamilton-Dutoit, Hendrik Vilstrup, Michael Sørensen

**Affiliations:** 1 Department of Hepatology and Gastroenterology, Aarhus University Hospital, Palle Juul Jensens Boulevard 99, 8200 Aarhus N, Denmark; 2 Department of Internal Medicine, Randers Regional Hospital, Skovlyvej 15, 8930 Randers, Denmark; 3 Department of Pathology, Aarhus University Hospital, Palle Juul Jensens Boulevard 99, 8200 Aarhus N, Denmark; 4 Department of Nuclear Medicine & PET, Aarhus University Hospital, Palle Juul Jensens Boulevard 99, 8200 Aarhus N, Denmark; 5 Department of Internal Medicine, Viborg Regional Hospital, Heibergs Alle 5A, 8800 Viborg, Denmark

**Correction to: BMC Gastroenterology (2022) 22:541**

**https://doi.org/10.1186/s12876-022-02574-6**

After publication of this article [1], the authors reported that (1) the main affiliation for Peter Lykke Eriksen should be affiliation 1 (not 2 as in the footnote on the title page); (2) the author name ‘Hendrik Vilstrup’ was incorrectly written as ‘DMSc Hendrik Vilstrup’; (3) in the Acknowledgements section Ms. Anne Kathrine Pedersen was missing.

The original article [1] has been corrected.

