# Supplementary material for: Experimental non-alcoholic fatty liver disease causes regional liver functional deficits as measured by the capacity for galactose metabolism while whole liver function is preserved
Source: BMC Gastroenterol. 2022 Dec 27;22:541. doi: 10.1186/s12876-022-02574-6 (PMC9793673; doi:10.1186/s12876-022-02574-6)
Supplement: Supplementary file 1 — Additional file 1: Supplementary Figures 1 and 2. [file 12876_2022_2574_MOESM1_ESM.pdf]

Supplementary material

Experimental non-alcoholic fatty liver disease causes regional liver functional deficits as measured by the capacity for galactose metabolism while whole liver function is preserved.

Peter Lykke Eriksen, Karen Louise Thomsen, Stephen Hamilton-Dutoit, Hendrik Vilstrup, Michael Sørensen

Department of Hepatology and Gastroenterology, Aarhus University Hospital
Correspondence: ple@clin.au.dk

BMC Gastroenterology

Supplementary figure 1: Body weight development

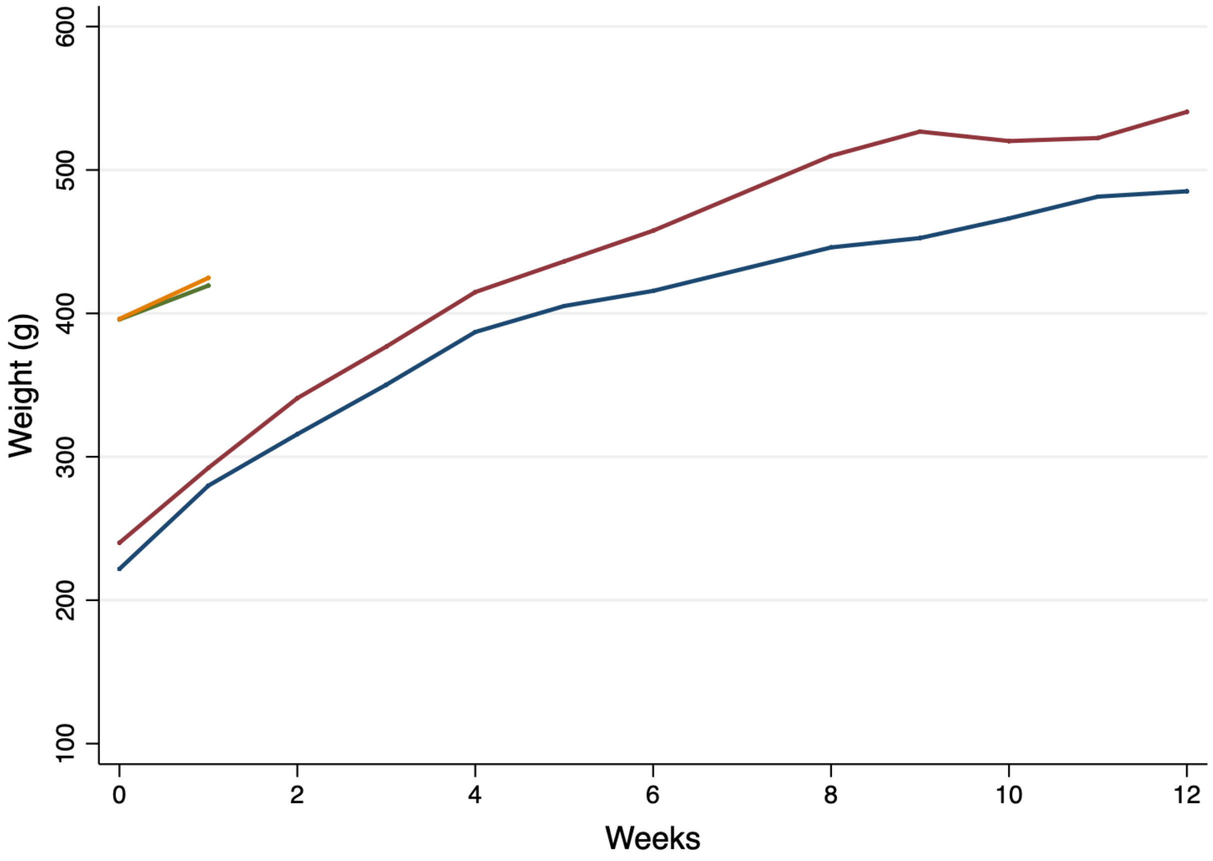

Rat body weight development from baseline (week 0) to the study day at week 12. High-fat high-cholesterol (HFHC) diet fed NAFLD rats 1 week (orange) and 12 weeks (red) and standard diet fed control rats 1 week (green) and 12 weeks (blue).

Supplementary figure 2: Liver accumulated ^{18}F-FDGal measured by PET and autoradiography

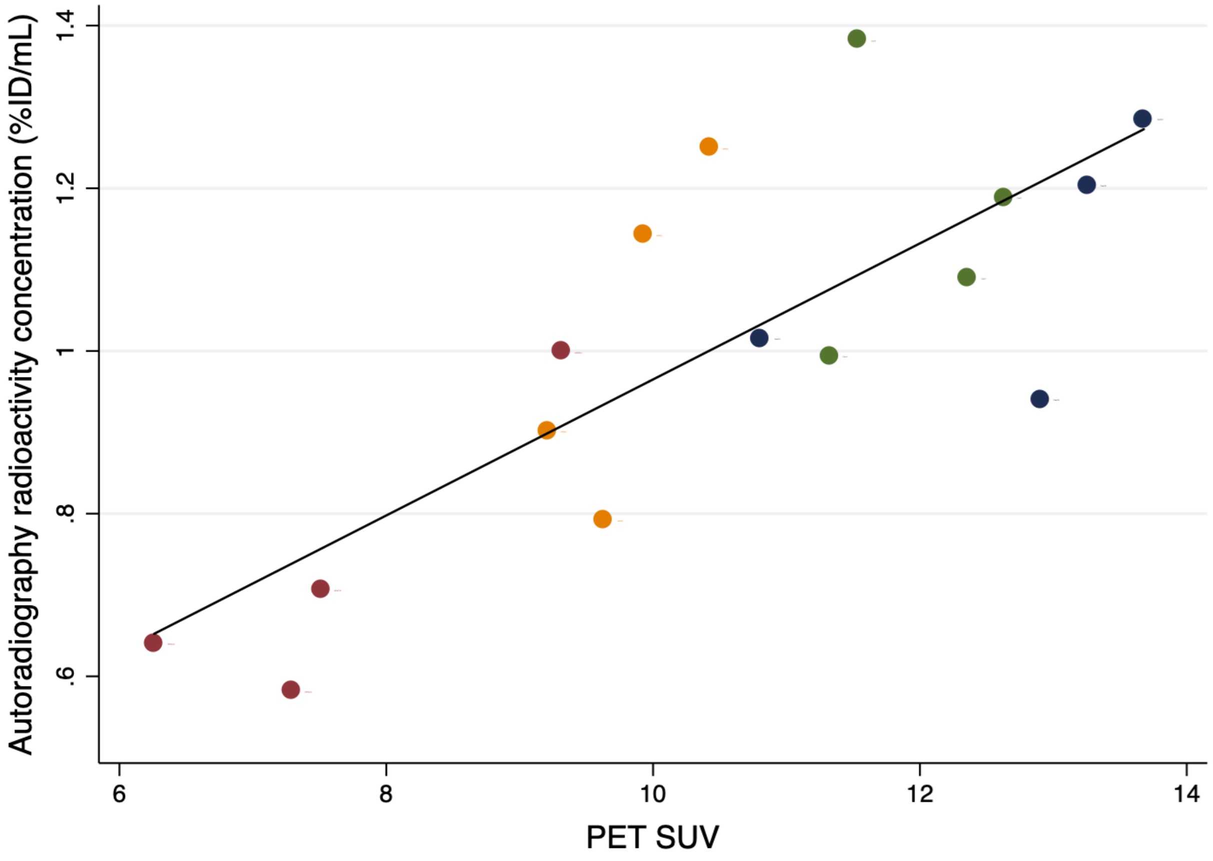

Relationship between liver accumulated ^{18}F-FDGal measured by micro positron emission tomography (PET) and expressed as standardaized uptake value (SUV) and by autoradiography with radioactivity concentration expressed as percentage of injected dose per mL liver tissue (%ID/mL liver tissue) in high-fat high-cholesterol (HFHC) diet fed NAFLD rats 1 week (orange) and 12 weeks (red) and standard diet red control rats 1 week (green) and 12 weeks (blue). The linear regression line is shown (Spearman's rank correlation coefficient, $\rho = 0.74$, $p = 0.001$).
